# Supplementary material for: Polyvinyl chloride degradation by a bacterium isolated from the gut of insect larvae
Source: Nat Commun. 2022 Sep 12;13:5360. doi: 10.1038/s41467-022-32903-y (PMC9468159; doi:10.1038/s41467-022-32903-y)

# SUPPLEMENTARY MATERIAL for

# Polyvinyl chloride degradation by a bacterium isolated from the gut of insect larvae

***Corresponding author:**

Dr. Feng Ju, Westlake University

Tel.: 571-87963205 (lab), 571-87380995 (office)

E-mail: jufeng@westlake.edu.cn; richieju520@gmail.com

**Supplementary Tables and Figure Legends**

**Supplementary Table 1 Detection and analysis results of additives in PVC film by GC-MS analysis**

**Supplementary Figure 1 Characterization of morphological changes of PVC film in the feces of *Spodoptera frugiperda* larva**

**Supplementary Figure 2 Screening and isolation of PVC film degrading strains and phylogenetic analysis of 16S rRNA gene sequence**

**Supplementary Figure 3 Degradation results of strain EMBL-1 on PVC film**

**Supplementary Figure 4 Spectrum of NMR experiments of PVC**

**Supplementary Figure 5 Detection results of degradation products of PVC film by GC-MS**

**Supplementary Figure 6 Detection and identification results of additives in PVC film and the degradation activity of strain EMBL-1s on main additives**

**Supplementary Figure 7 The whole-genome map of strain EMBL-1**

**Supplementary Figure 8 Results of proteome experiments and expression purification of catalase-peroxdiase**

**Supplementary Figure 9 Physicochemical characterization of PVC degradation by**

**catalase-peroxidase**

**Supplementary Figure 10 Potential degradation products detection of PVC by catalase-peroxidase**

## Supplementary Tables and Figures

**Supplementary Table 1 Detection and analysis results of additives in PVC film by GC-MS analysis**

| Name | RT/min | Qualitative and quantitative transition | Standard curve/$R^2$ | Content in PVC film (%) |
|---|---|---|---|---|
| DOA | 11.78 | 129,57,112/129 | y=2E+06x-226835, $R^2$=0.9925 | 22.90 |
| DOTP | 14.53 | 70,112,149/70 | y=952890x-2E+06, $R^2$=0.9935 | 5.23 |
| Erucylamide | 15.09 | 59,72,55/59 | y=523400x-1E+06, $R^2$=0.9936 | 2.05 |

**Supplementary Figure 1 Characterization of morphological changes of PVC film in the feces of *Spodoptera frugiperda* larva**

a, Scanning electron microscopy (SEM) results showing clear degradation of PVC film recovered from larval feces in PVC group. b, SEM results showing no visible biodegradation of PVC film recovered from feces in Antibiotic group (in which gentamicin antibiotic was used to inactivate most gut microbes of the larva). For both a and b, subfigure (1) to (4) represented the degradation of PVC film from low magnification to high magnification in SEM. At least 3 times experiment was repeated independently.

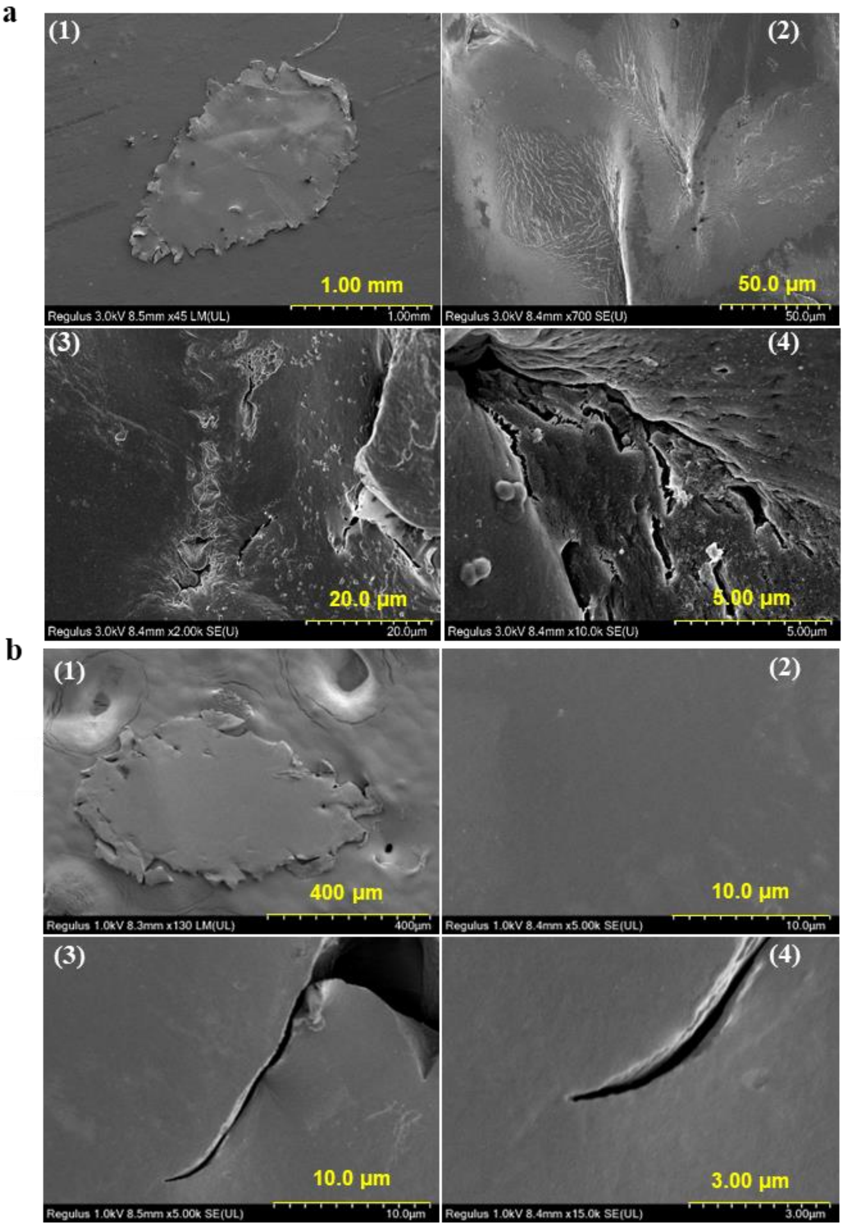

**Supplementary Figure 2 Screening and isolation of PVC film degrading strains and phylogenetic analysis of 16S rRNA gene sequence**

a, Experimental screening and isolation (The dissected intestine of larva in PVC group was used to inoculate and enrich for degrading strain EMBL-1, which was cultured on PVC film in MSM liquid medium and LB solid medium). b, Phylogenetic tree of 16S rRNA gene sequences showing PVC-degrading strain EMBL-1 as a novel Klebsiella strain most closely related to *Klebsiella variicola* and *Klebsiella pneumoniae* (The analysis was conducted using the Fast Minimum Evolution method at a maximum sequence difference of 0.75).

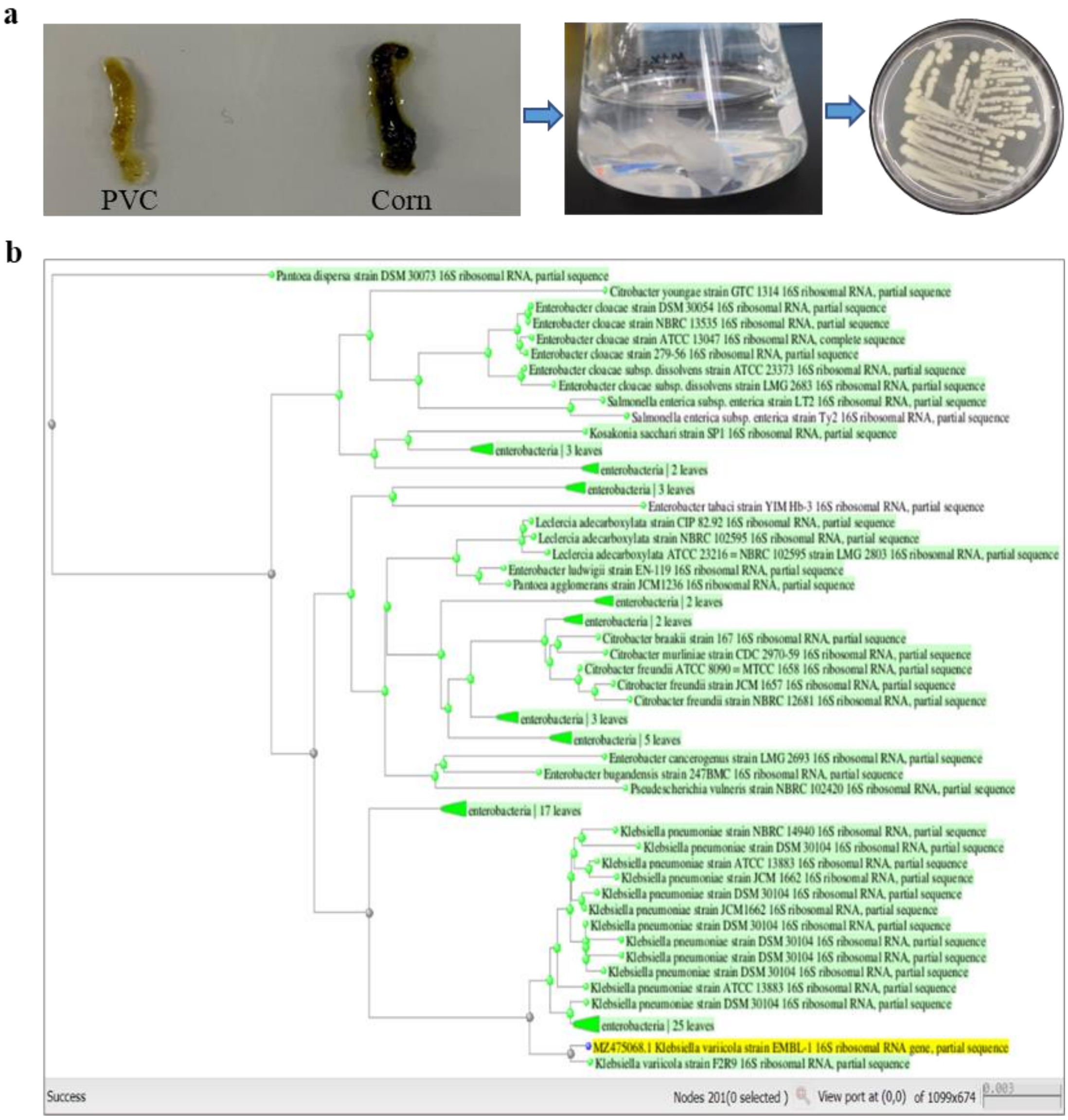

**Supplementary Figure 3 Degradation results of strain EMBL-1 on PVC film**

a, SEM images of the cracks and pits formed on the PVC film by strain EMBL-1 on day 90. At least 3 times experiment was repeated independently. b, Water contact angle of PVC film after co-culturing with strain EMBL-1 significantly decreased compared with the control group (t-test $P < 0.05$, n = 3 samples each). c, Tensile strength of PVC film in the control group and the EMBL-1 group after 90 days. d, $OD_{600}$ values during 65-day growth of strain EMBL-1 using soybean oil (SO) as the sole organic carbon substrate, which showed no growth of strain EMBL-1 compared with the control groups. Each group had 3 replicates and the mean values were visualized. e-f, Thermal gravimetric analysis (TGA) results of PVC film in control and EMBL-1 groups during 90 days. g, FTIR results of PVC film in control groups during 90 days. Source data are provided as a Source Data file.

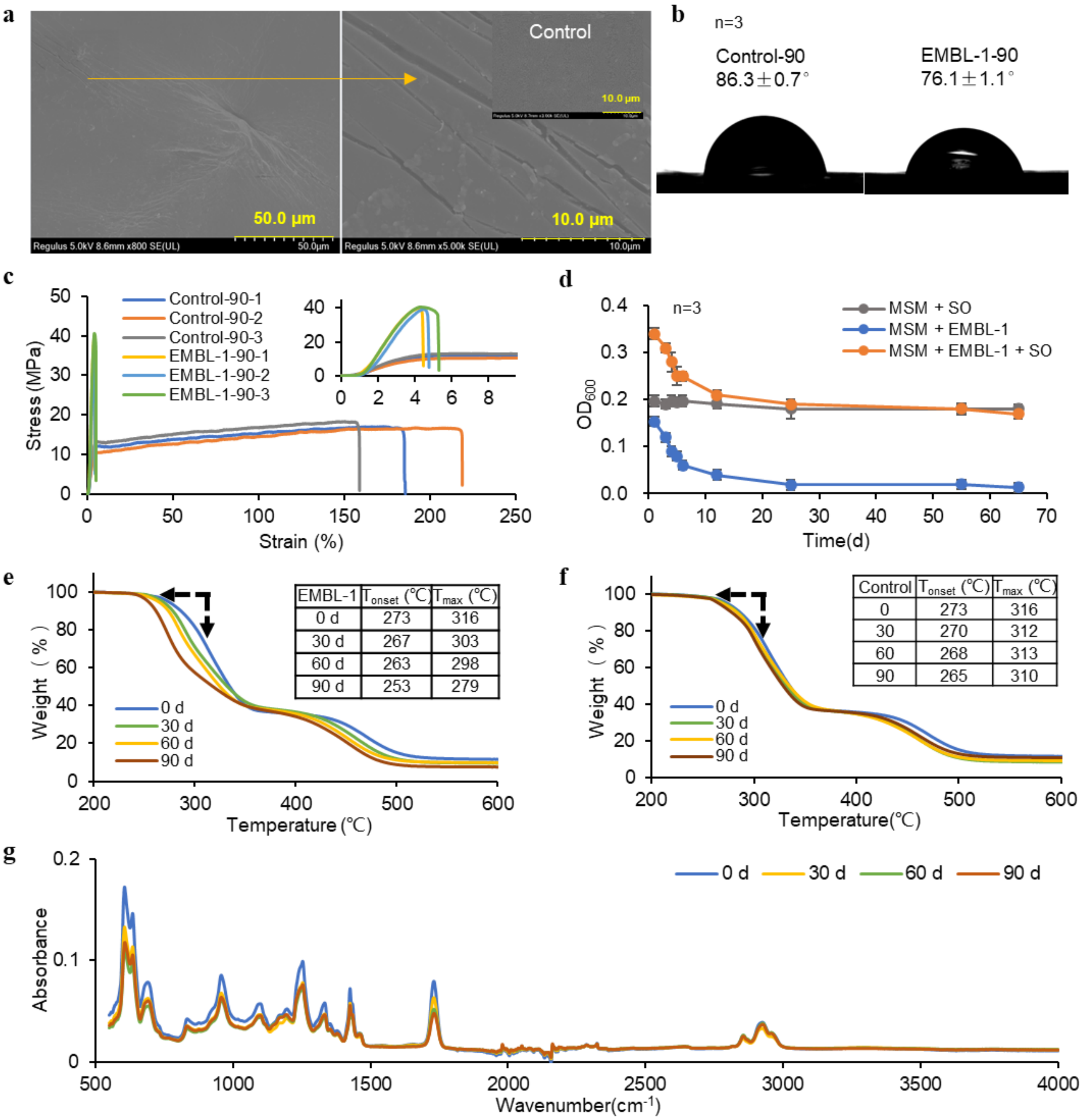
a
Control
50.0 μm
10.0 μm
10.0 μm
Regulus 5.0kV 8.6mm x800 SE(UL)
50.0μm
Regulus 5.0kV 8.6mm x5.00k SE(UL)
10.0μm
b
n=3
Control-90
86.3±0.7°
EMBL-1-90
76.1±1.1°
c
Control-90-1
Control-90-2
Control-90-3
EMBL-1-90-1
EMBL-1-90-2
EMBL-1-90-3
Stress (MPa)
Strain (%)
d
n=3
MSM + SO
MSM + EMBL-1
MSM + EMBL-1 + SO
OD600
Time(d)
e
Weight （%）
Temperature (℃)
0 d
30 d
60 d
90 d
EMBL-1 | Tonset (℃) | Tmax (℃)
0 d | 273 | 316
30 d | 267 | 303
60 d | 263 | 298
90 d | 253 | 279
f
Weight （%）
Temperature(℃)
0 d
30 d
60 d
90 d
Control | Tonset (℃) | Tmax (℃)
0 | 273 | 316
30 | 270 | 312
60 | 268 | 313
90 | 265 | 310
g
Absorbance
Wavenumber(cm-1)
0 d
30 d
60 d
90 d

62

**Supplementary Figure 4 Spectrum of NMR experiments of PVC**

a, DOSY Spectrum of pure PVC in control and EMBL-1 group on 90 days. b, 2D $^{1}H$-$^{1}H$ COSY spectrum of pure PVC in EMBL-1 group on 90 days. c, 2D $^{1}H$-$^{1}H$ NOESY spectrum of pure PVC in EMBL-1 group on 90 days. d, 2D $^{1}H$-$^{13}C$ HSQC spectrum of pure PVC in EMBL-1 group on 90 days. e, 2D $^{1}H$-$^{13}C$ HMBC spectrum of pure PVC in EMBL-1 group on 90 days.

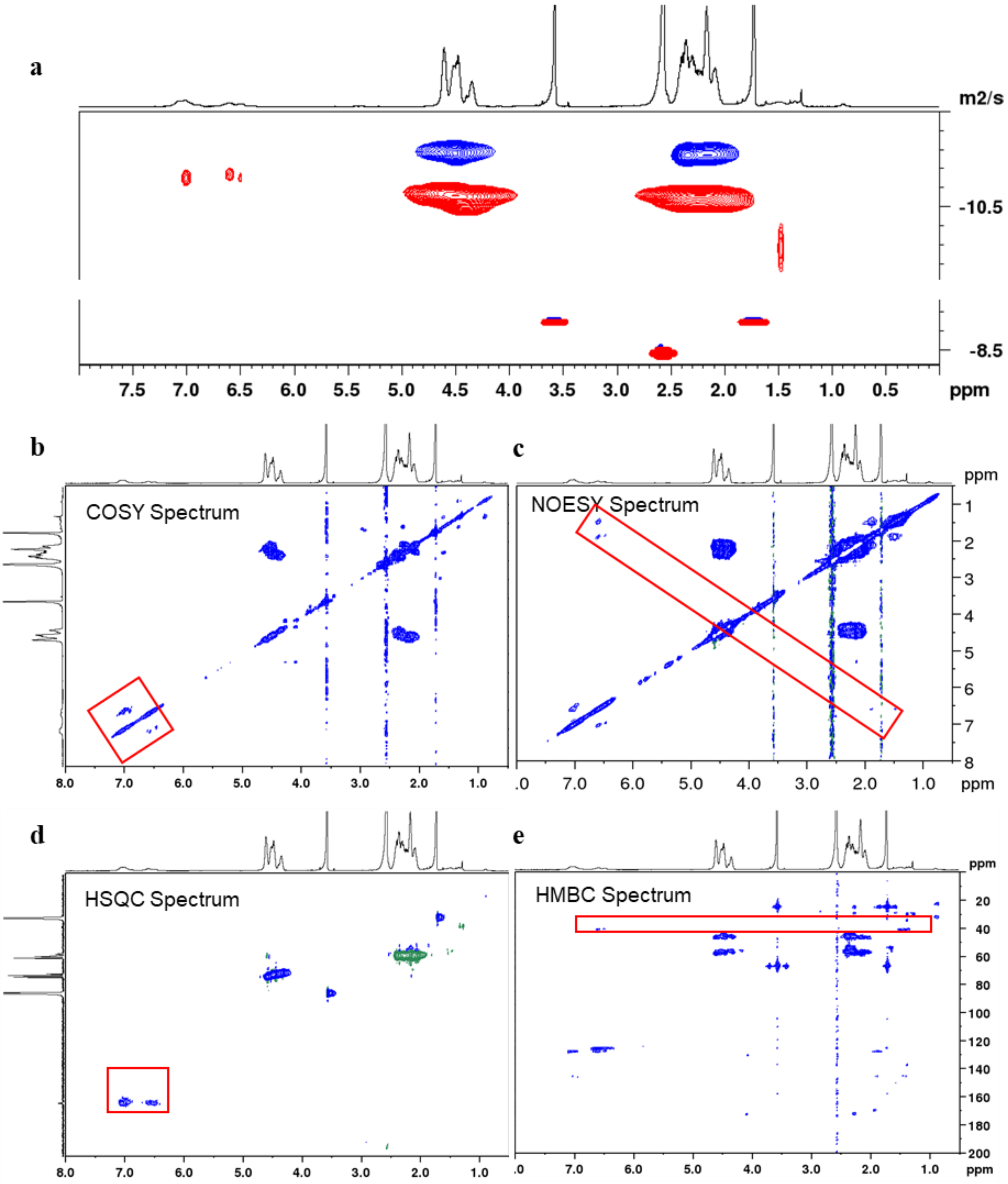

**Supplementary Figure 5 Detection results of degradation products of PVC film by GC-MS**

a, the TIC diagram of degradation products of PVC film in two groups by the time of 90 d. b-f, the mass spectrum and structural formula of the potential degradation products 1-5.

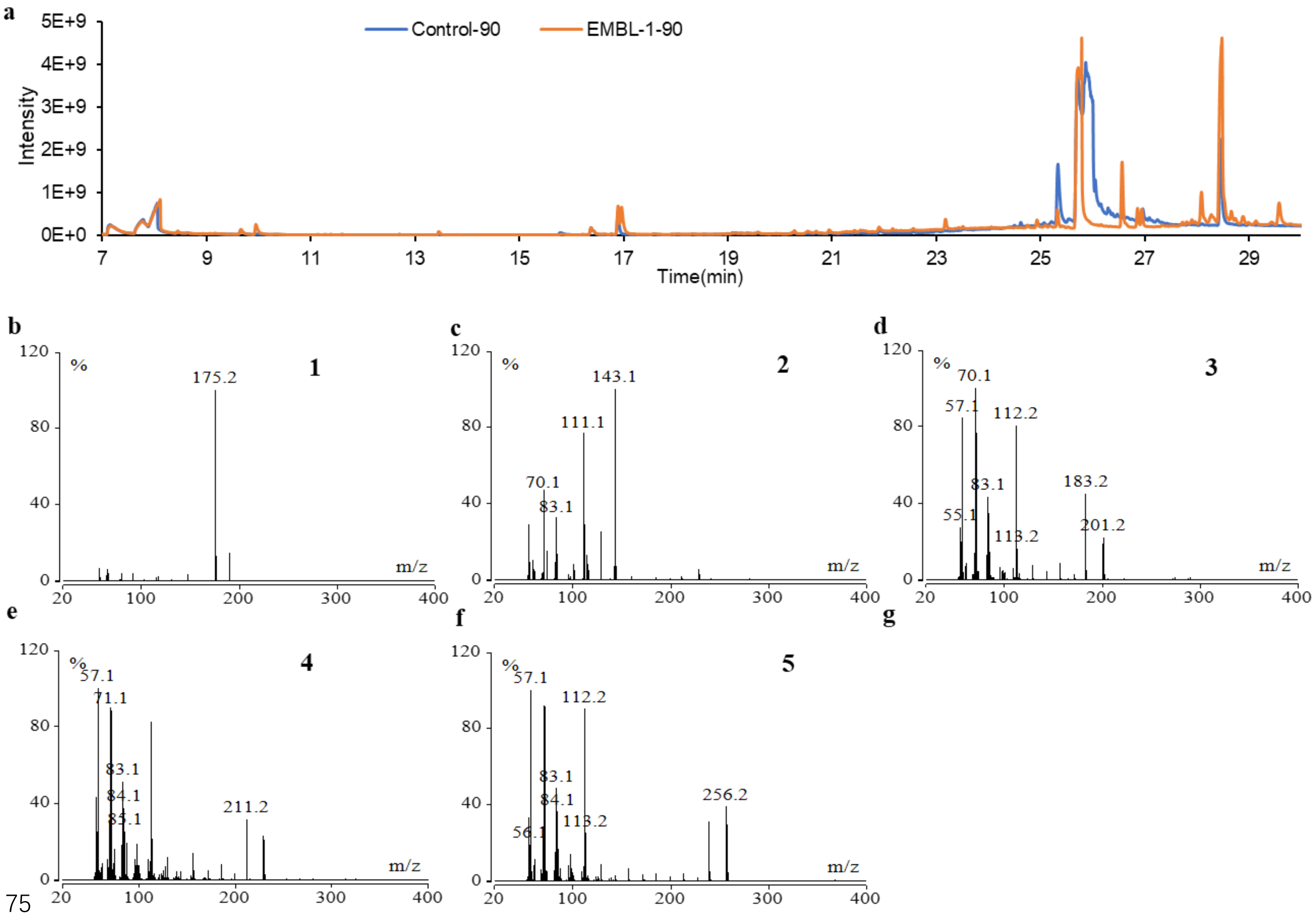

**Supplementary Figure 6 Detection and identification results of additives in PVC film and the degradation activity of strain EMBL-1 on main additives**

a, the Py/GC-MS diagram of three additives in PVC film. b, the TIC diagram of three additives identification in PVC film. c-e, the mass spectrum of three additives. f, the result of degradation activity of strain EMBL-1 on three additives. The experiments were conducted in triplicates, and mean value ± standard deviation was visualized.

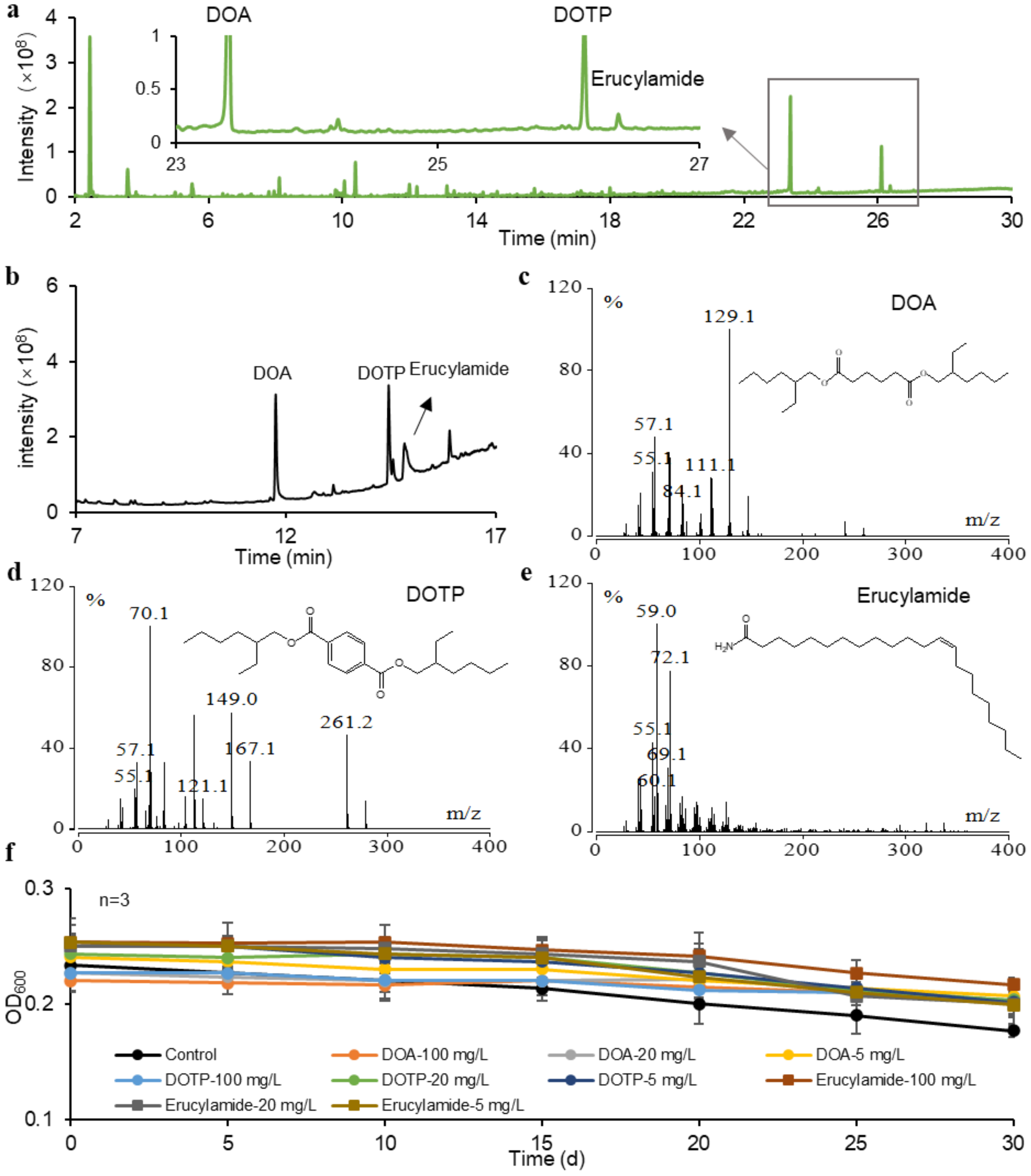

**Supplementary Figure 7 The whole-genome map of strain EMBL-1**

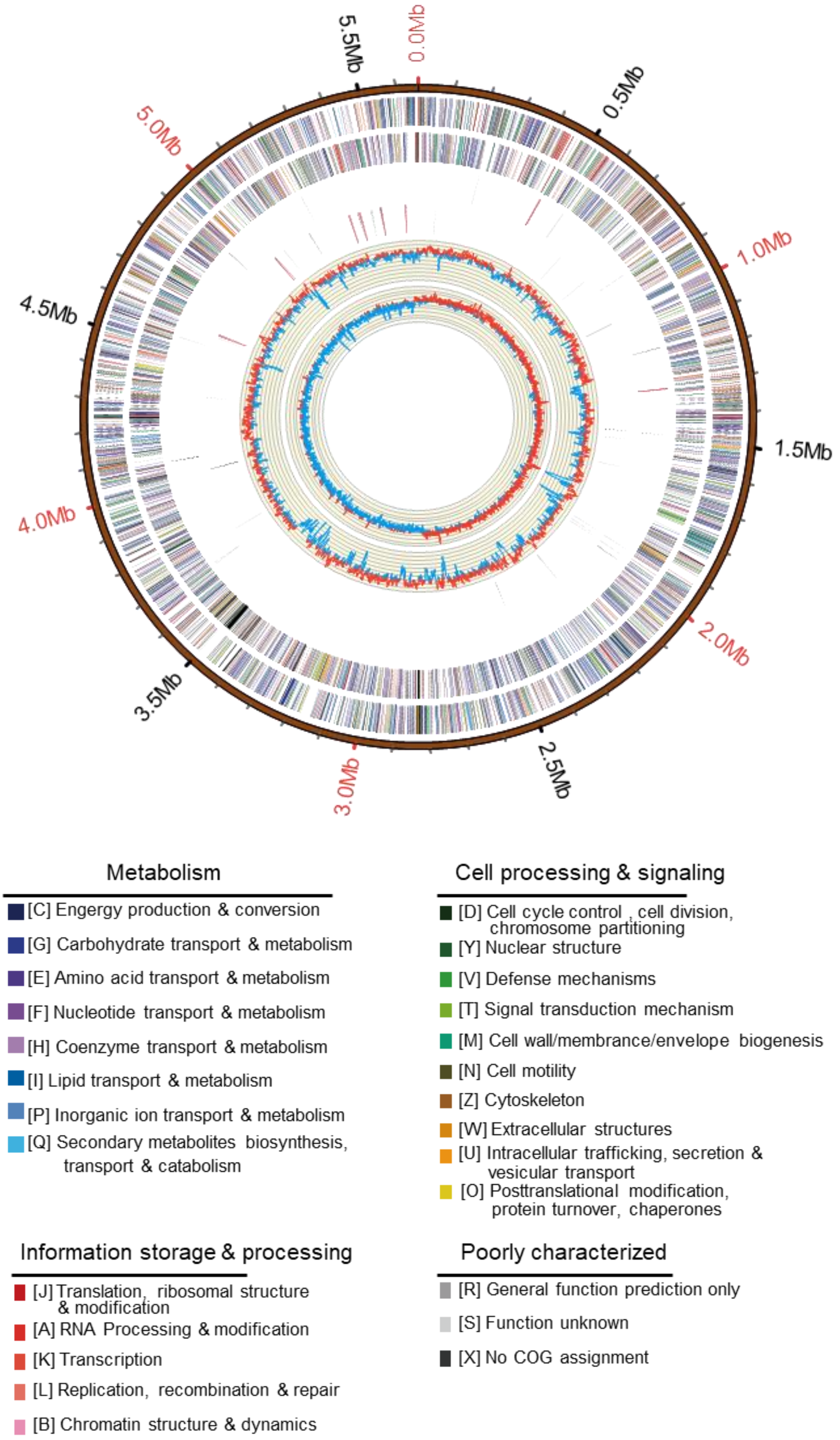

**Supplementary Figure 8 Results of proteome experiments and expression purification of catalase-peroxdiase**

a, Growth curve of strain EMBL-1 under different carbon source conditions. b, The concentration of proteins in four groups detected by Bradford method. c, Construction results of expression strain of catalase-peroxdiase (M means protein marker,1 means the strain lysate before induction, 2 means the strain lysate after induction ). d, purification results of catalase-peroxdiase using Ni-IDA Agarose Magnetic Beads (M means protein marker,1 means the strain lysate after induction, 2-3 mean the eluention during purification, 4 means the eluention with catalase-peroxdiase). For a and b, the experiments were conducted in triplicates, and mean value ± standard deviation was visualized. For c and d, the experiments were repeated at least 3 times independently. Source data were provided as a Source Data file.

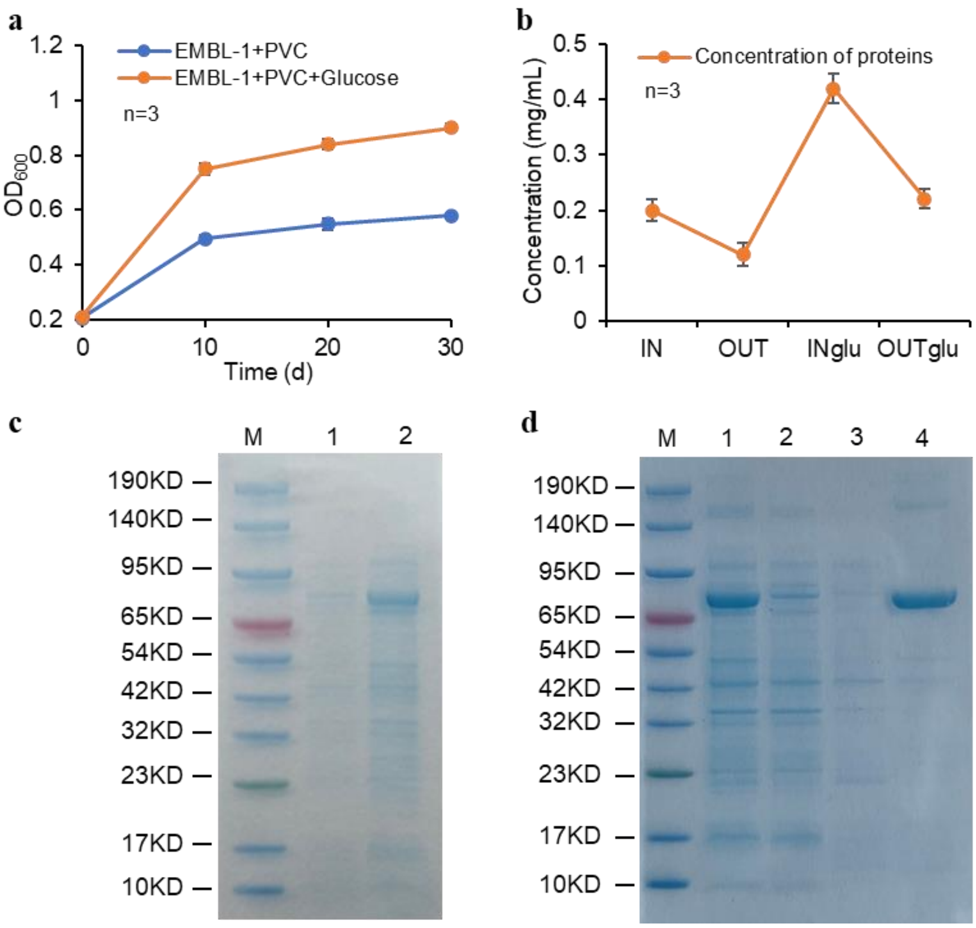

**Supplementary Figure 9 Physicochemical characterization of PVC degradation by catalase-peroxidase**

a, FTIR results for PVC in the PVC-Cp group and PVC control group. b, Molecular weight of PVC in the PVC-catalase-peroxidase group and PVC control group. The experiments were conducted in triplicates and mean value ± standard deviation was visualized. Source data were provided as a Source Data file.

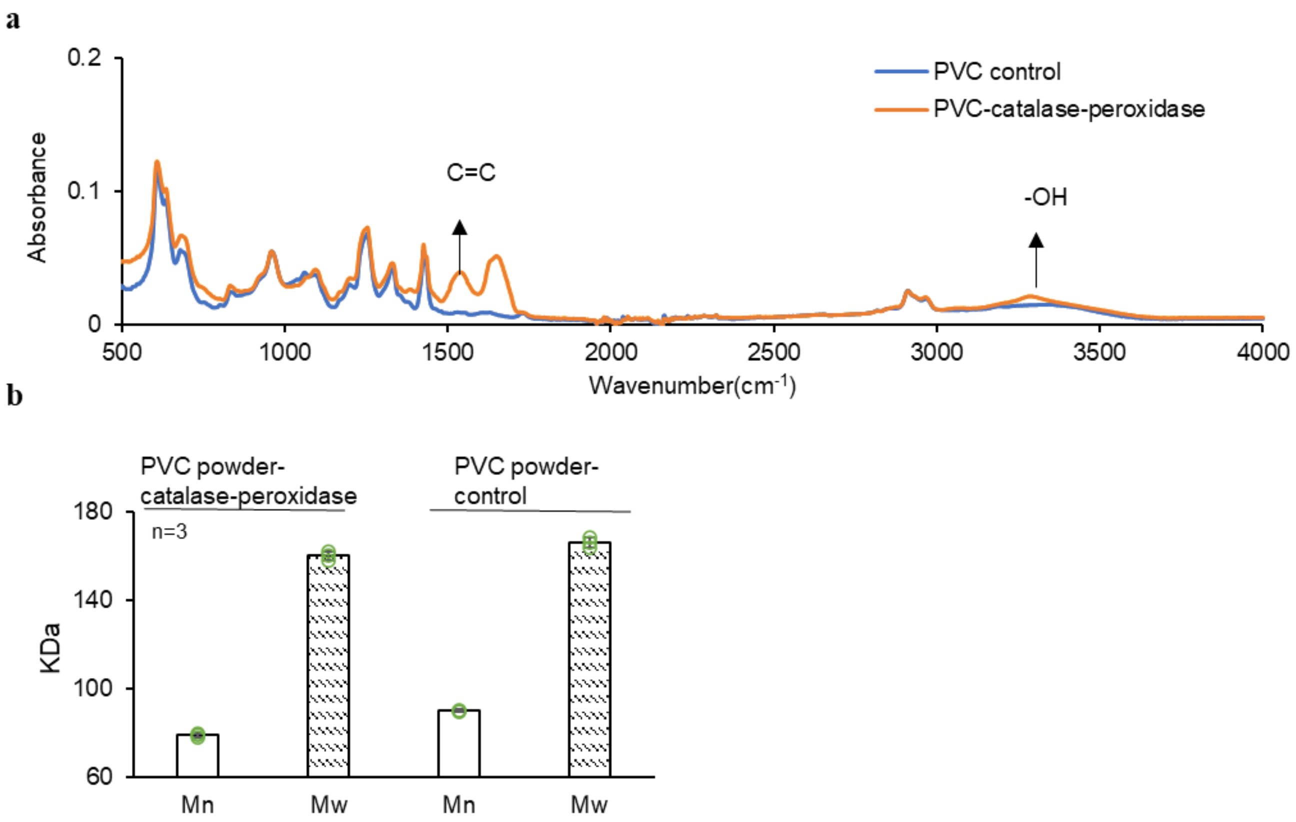

**Supplementary Figure 10 Potential degradation products detection of PVC by catalase-peroxidase**

a, The detection results of GC-MS of degradation products from PVC-catalase-peroxidase group and PVC control group. b, the mass spectrum and structural formula of the potential degradation products.

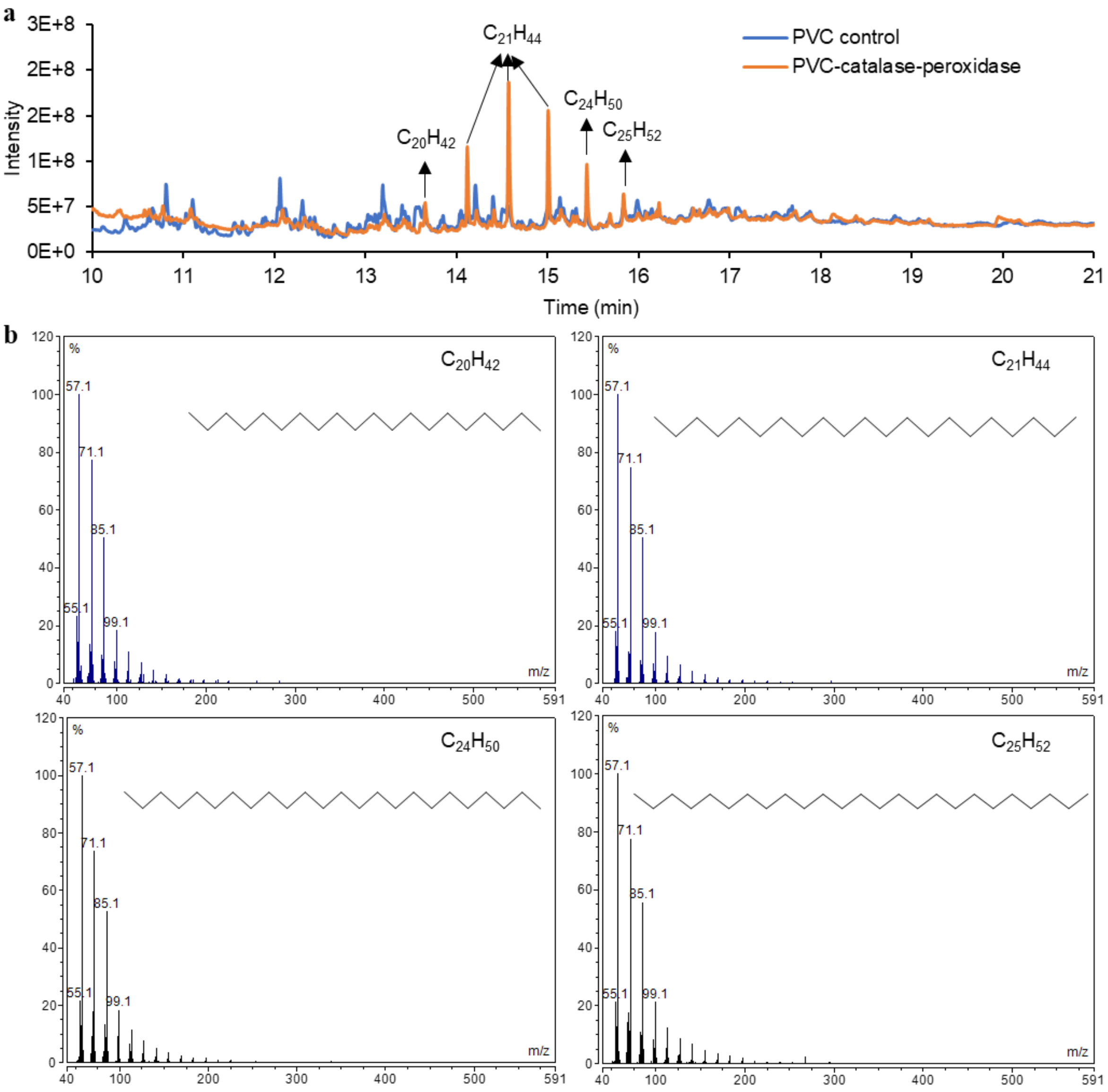

Supplement: Supplementary file 1 — Supplementary information [file 41467_2022_32903_MOESM1_ESM.pdf]
