# Supplementary material for: Polyvinyl chloride degradation by a bacterium isolated from the gut of insect larvae
Source: Nat Commun. 2022 Sep 12;13:5360. doi: 10.1038/s41467-022-32903-y (PMC9468159; doi:10.1038/s41467-022-32903-y)
Supplement: Supplementary file 3 — Description of Additional Supplementary Files [file 41467_2022_32903_MOESM3_ESM.pdf]

Title: Supplementary Data 1.
Description: Annotated results of the genome sequencing data of EMBL-1 strain。

Title: Supplementary Data 2.
Description: The analysis data of mass spectrometry of proteins of different treatment groups. Reference genome (Klebsiella variicola 118)

Title: Supplementary Data 3.
Description: The transcriptome sequencing analysis data of EMBL-1 strain degrading PVC film under different culture conditions (Reference genome (Klebsiella variicola FH-1), up-regulated genes of EMBL-1 genomes in the group with PVC film as the only carbon source)
