# Supplementary material for: Polyvinyl chloride degradation by a bacterium isolated from the gut of insect larvae
Source: Nat Commun. 2022 Sep 12;13:5360. doi: 10.1038/s41467-022-32903-y (PMC9468159; doi:10.1038/s41467-022-32903-y)
Supplement: Supplementary file 7 — Reporting Summary [file 41467_2022_32903_MOESM7_ESM.pdf]

nature portfolio

Corresponding author(s): Feng Ju

Last updated by author(s): Aug 8, 2022

Reporting Summary

Nature Portfolio wishes to improve the reproducibility of the work that we publish. This form provides structure for consistency and transparency in reporting. For further information on Nature Portfolio policies, see our Editorial Policies and the Editorial Policy Checklist.

Statistics

For all statistical analyses, confirm that the following items are present in the figure legend, table legend, main text, or Methods section.

n/a	Confirmed	
☐	☒	The exact sample size (*n*) for each experimental group/condition, given as a discrete number and unit of measurement
☐	☒	A statement on whether measurements were taken from distinct samples or whether the same sample was measured repeatedly
☐	☒	The statistical test(s) used AND whether they are one- or two-sided<br>*Only common tests should be described solely by name; describe more complex techniques in the Methods section.*
☐	☒	A description of all covariates tested
☐	☒	A description of any assumptions or corrections, such as tests of normality and adjustment for multiple comparisons
☐	☒	A full description of the statistical parameters including central tendency (e.g. means) or other basic estimates (e.g. regression coefficient) AND variation (e.g. standard deviation) or associated estimates of uncertainty (e.g. confidence intervals)
☐	☒	For null hypothesis testing, the test statistic (e.g. *F*, *t*, *r*) with confidence intervals, effect sizes, degrees of freedom and *P* value noted<br>*Give P values as exact values whenever suitable.*
☒	☐	For Bayesian analysis, information on the choice of priors and Markov chain Monte Carlo settings
☒	☐	For hierarchical and complex designs, identification of the appropriate level for tests and full reporting of outcomes
☒	☐	Estimates of effect sizes (e.g. Cohen's *d*, Pearson's *r*), indicating how they were calculated

Our web collection on statistics for biologists contains articles on many of the points above.

Software and code

Policy information about availability of computer code

Data collection	An FTIR microspectrometer (Thermo Fisher, Nicolet iS50, China) with a scan range of 4000–500 cm-1 using OMNIC (v 9.10.928) software was used to analyze and detect the changes in the surface chemical composition and functional groups of the PVC film (32 scans for each spectrum).The spectra of NMR experiments data were analyzed using MestReNova software (version 12.0.0).The mass spectrometer of proteins was operated in the data-dependent acquisition mode using the Xcalibur 4.1 software and there is a single full-scan mass spectrum in the Orbitrap (300-1800 m/z, 60,000 resolution) followed by 20 data-dependent MS/MS scans at 30% normalized collision energy. Each mass spectrum was analyzed using the Peak studio for the database searching.
Data analysis	For the 16S rRNA gene amplicon data analysis, FastQC (v0.11.9) and cutadapt (v1.18) were first used to check the quality of the raw data and excise double-ended primers (fastaq files). Dada2 (v1.14) was then used to cluster the input sequence with default parameter settings and for further denoising after importing double-ended data through Quantitative Insight into Microbial Ecology (QIIME2-2020.6) and input-format setting parameters. The next step was to select high-quality areas based on FastQC's report results. Taxonomic classification was conducted using the qiime2 built-in package, and the feature-classifier classify-sklearn machine learning method was used for taxonomic annotation using the SILVA 138 SSU as the reference database. The generated files were imported into R studio version 1.1.414 (R version 4.0.3), and phyloseq (v1.32.0) was used for statistical analysis and visualization of the data. In addition, the survival (v3.2.7) and survminer packages (v0.4.9) were used to calculate and draw the survival curve, while ggplot2 (v3.3.3) was used to draw box plots of weight. ANOVA was used to check the significance of differences between experimental groups. The bioinformatic analysis of whole-genome sequencing of EMBL-1 included five major steps: raw data quality control, genome assembly, genome component analysis, functional annotation, and genome visualization. In brief, the quality control of raw short reads from Illumina sequencing and raw long reads from Nanopore sequencing were performed in Fastp 0.19.5 and Mecat 2, respectively. Then, the clean short and long reads were coassembled to reconstruct complete genomes using Unicycle (https://github.com/rrwick/Unicycler) to generate complete sequences. The coding sequences (CDSs) were predicted using Glimmer version 3.02 59. Databases such as KEGG, COG, GO, and CAZy were used for functional annotation. In addition, MUMmer

software (v3.23) was used to compare the target genome with the reference genome to determine the collinearity between the genomes. The raw transcriptomic reads of were first filtered using fastp to remove the reads that contained 10 low-quality bases (base quality score less than 20) or lengths shorter than 36 bp. Then, the resulting high-quality (HQ) reads were aligned to the K. variicola reference genome (K. variicola strain FH-1) using hisat2. After alignment, the read counts for each gene were extracted using htseq-count. The gene expression profiles of triplicate transcriptomes in the two groups were compared with PCoA, which inspected one outlier dataset in each group (due to unexpected experimental errors) that was discarded from downstream analysis. Differential expression (DE) at the gene level in our two groups (group a and group b) was evaluated using edgeR version 3.30.3, implemented in R 4.0.3. The p-values presented were adjusted for multiple testing with the procedure of Benjamini and Hochberg to control the type I error rate, and a cutoff of $p \le 0.05$ was used as a threshold to define differential expression. Kraken2 was used to check for contamination in the RNA-seq data.

For manuscripts utilizing custom algorithms or software that are central to the research but not yet described in published literature, software must be made available to editors and reviewers. We strongly encourage code deposition in a community repository (e.g. GitHub). See the Nature Portfolio guidelines for submitting code & software for further information.

Data

Policy information about availability of data

All manuscripts must include a data availability statement. This statement should provide the following information, where applicable:

- Accession codes, unique identifiers, or web links for publicly available datasets
- A description of any restrictions on data availability
- For clinical datasets or third party data, please ensure that the statement adheres to our policy

The 16S rRNA gene amplicon sequence and complete genome sequence of strain EMBL-1 strain generated in this study have been deposited in the National Center for Biotechnology Information (NCBI) database under accession code MZ475068 and CP079802, respectively. The raw transcriptomic data were uploaded to the National Center for Biotechnology Information (NCBI) database under accession code PRJNA866083. The mass spectrometry proteomics data have been deposited to the ProteomeXchange Consortium via the PRIDE partner repository with the dataset identifier PXD035850. Source data are provided with this paper.

Human research participants

Policy information about studies involving human research participants and Sex and Gender in Research.

Reporting on sex and gender	no human research
Population characteristics	no human research
Recruitment	no human research
Ethics oversight	no human research

Note that full information on the approval of the study protocol must also be provided in the manuscript.

Field-specific reporting

Please select the one below that is the best fit for your research. If you are not sure, read the appropriate sections before making your selection.

☐ Life sciences ☐ Behavioural & social sciences ☒ Ecological, evolutionary & environmental sciences

For a reference copy of the document with all sections, see nature.com/documents/nr-reporting-summary-flat.pdf

Ecological, evolutionary & environmental sciences study design

All studies must disclose on these points even when the disclosure is negative.

Study description	We hypothesized that the intestinal microbiota of S. frugiperda larva should play an essential role in digesting PVC film, which enabled the observed and experimentally verified larval survival on PVC film. To test this hypothesis and validate the larval ability to live on PVC film, 130 4th-instar larvae with the same growth status were divided into four groups: 1) the control group (starvation, 15 pcs), 2) the Corn group (fed corn leaves, 35 pcs), 3) the PVC group (fed PVC film, 50 pcs), and 4) the Atibiotic group (fed corn leaves with gentamicin for 3 d, and then PVC film, 30 pcs) . The body weight of all the numbered larvae were measured after 24 h of starvation and after the 5-day experiment. By the end of the experiment, the excreted feces of each experimental group were collected, and the number of survivors was counted. The numbers of culturable cells in the intestinal microbiota in Antibiotic group and Corn group were also determined. Subsequently, the surviving larvae in the Corn group and PVC group were dissected under aseptic conditions to obtain intestinal samples.The intestines of every 10 larva in the Corn group or PVC groups were combined and counted as one replicate sample. Three replicates in each group were used for intestinal microbial DNA extraction and sequencing, which were labeled and temporarily stored at 4 ℃ until further operation.
Research sample	During in-lab cultivation of an agriculturally invasive pest Spooptera frugiperda, we accidentally found that the larvae could bite and digest the plastic film of PVC for survival.To test this hypothesis and validate the larval ability to live on PVC film, 130 4th-instar larvae we breeding with the same growth status were used to conduct research.

Sampling strategy	Under the condition of 23 ℃, the larva of Spodoptera frugiperda can be divided into six instars, with a duration of 15-16 days, of which the first instar lasts for 3 days, the second instar lasts for 2 days, the third instar lasts for 2 days, the fourth instar lasts for 2 days, the fifth instar lasts for 2-3 days, and the sixth instar lasts for 4-5 days. We chose the 4th instar larvae for the experiment because they have a stable gut microbial flora and good growth status at this stage.The whole experiment lasted for 5 days, which was a reasonable time.
Data collection	The body weight of all the numbered larvae were measured with an electronic balance of 1 / 10000 after 24 h of starvation and after the 5-day experiment. By the end of the experiment, the excreted feces of each experimental group were collected by breeder, and the number of survivors was counted by breeder. The numbers of culturable cells in the intestinal microbiota in Antibiotic group and Corn group were also determined and counted by breeder. Because the number of larvae was not too much and the data statistics are simple, the collection of these data was recorded manually with paper and pen.
Timing and spatial scale	The entire experiment lasted for 5d in Dec 2020 (12th-16th Dec).The body weight of all the numbered larvae were measured at the begaining and ending of the experiment. Count the number of dead larvae every ninght.
Data exclusions	no data were exclude from the analyses.
Reproducibility	the experiments were replicated two months, all attempts to repeat the experiment were successful.
Randomization	all the active 4th-instar larva were allocated to four groups randomly.
Blinding	In the study, we only use random allocation at the beginning of larval grouping. After allocation, different groups of larvae will be given different treatments (different food or no food), so we know the treatment conditions in the data collection and statistics . Therefore, there is no blinding in data statistics and processing.

Did the study involve field work? ☐ Yes ☒ No

Reporting for specific materials, systems and methods

We require information from authors about some types of materials, experimental systems and methods used in many studies. Here, indicate whether each material, system or method listed is relevant to your study. If you are not sure if a list item applies to your research, read the appropriate section before selecting a response.

Materials & experimental systems

n/a	Involved in the study
☒	☐ Antibodies
☒	☐ Eukaryotic cell lines
☒	☐ Palaeontology and archaeology
☐	☒ Animals and other organisms
☒	☐ Clinical data
☒	☐ Dual use research of concern

Methods

n/a	Involved in the study
☒	☐ ChIP-seq
☒	☐ Flow cytometry
☒	☐ MRI-based neuroimaging

Animals and other research organisms

Policy information about studies involving animals; ARRIVE guidelines recommended for reporting animal research, and Sex and Gender in Research

Laboratory animals	no laboratory animals were used in the study.
Wild animals	Spodoptera frugiperda is a moth of the genus Spodoptera in the family Spodoptera. 3-4th instar larva were captured using tweezers from corn leaves in the corn field and took back to lab in a breathable box with corn leaves inside. For the larvae used in the experiment, the living larvae continue to be raised normally for subculture and the dead larvae are buried after the end of the experiment.
Reporting on sex	larva of Spodoptera frugiperda were neutral.
Field-collected samples	The larva of Spodoptera frugiperda were breed in an artificial climate room (temperature 22℃, humidity 55%, 8h of light and 16 h of darkness. All larvae were only used for the experiment, and the breeding of larvae was stopped after the experiment.
Ethics oversight	no ethical approval or guidance was required.

Note that full information on the approval of the study protocol must also be provided in the manuscript.
